# The rise of mortality from mental and neurological diseases in Europe, 1979–2009: observational study

**DOI:** 10.1186/1471-2458-14-840

**Published:** 2014-08-13

**Authors:** Johan P Mackenbach, Marina Karanikolos, Caspar WN Looman

**Affiliations:** Department of Public Health, Erasmus MC, P.O. Box 2040, 3000 CA Rotterdam, Netherlands; European Centre on Health of Societies in Transition, London School of Hygiene and Tropical Medicine, London, UK

**Keywords:** Mortality, Mental and behavioural disorders, Diseases of the nervous system, Dementias, Psychoactive substance use, Meningitis, Parkinson’s disease, Alzheimer’s disease, Multiple sclerosis, Epilepsy, World Value Survey, Europe

## Abstract

**Background:**

We studied recent trends in mortality from seven mental and neurological conditions and their determinants in 41 European countries.

**Methods:**

Age-standardized mortality rates were analysed using standard methods of descriptive epidemiology, and were related to cultural, economic and health care indicators using regression analysis.

**Results:**

Rising mortality from mental and neurological conditions is seen in most European countries, and is mainly due to rising mortality from dementias. Mortality from psychoactive substance use and Parkinson’s disease has also risen in several countries. Mortality from dementias has risen particularly strongly in Finland, Iceland, Malta, Netherlands, Spain, Sweden and the United Kingdom, and is positively associated with self-expression values, average income, health care expenditure and life expectancy, but only the first has an independent effect.

**Conclusions:**

Although trends in mortality from dementias have probably been affected by changes in cause-of-death classification, the high level of mortality from these conditions in a number of vanguard countries suggests that it is now among the most frequent causes of death in high-income countries. Recognition of dementias as a cause of death, and/or refraining from life-saving treatment for patients with dementia, appear to be strongly dependent on cultural values.

**Electronic supplementary material:**

The online version of this article (doi:10.1186/1471-2458-14-840) contains supplementary material, which is available to authorized users.

## Background

Over the past decades, mortality has declined substantially in many European countries, as a result of declines for many specific causes of death, particularly cardiovascular disease. This has been interpreted by some as a new stage in the epidemiologic transition, in which the mean age of death shifts upwards [1], and the cause-of-death pattern shifts towards diseases of very old age such as dementias [2, 3]. Some early authors have even warned for a “pandemic of mental disorders and disabilities” [4, 5].

**Figure 1:**
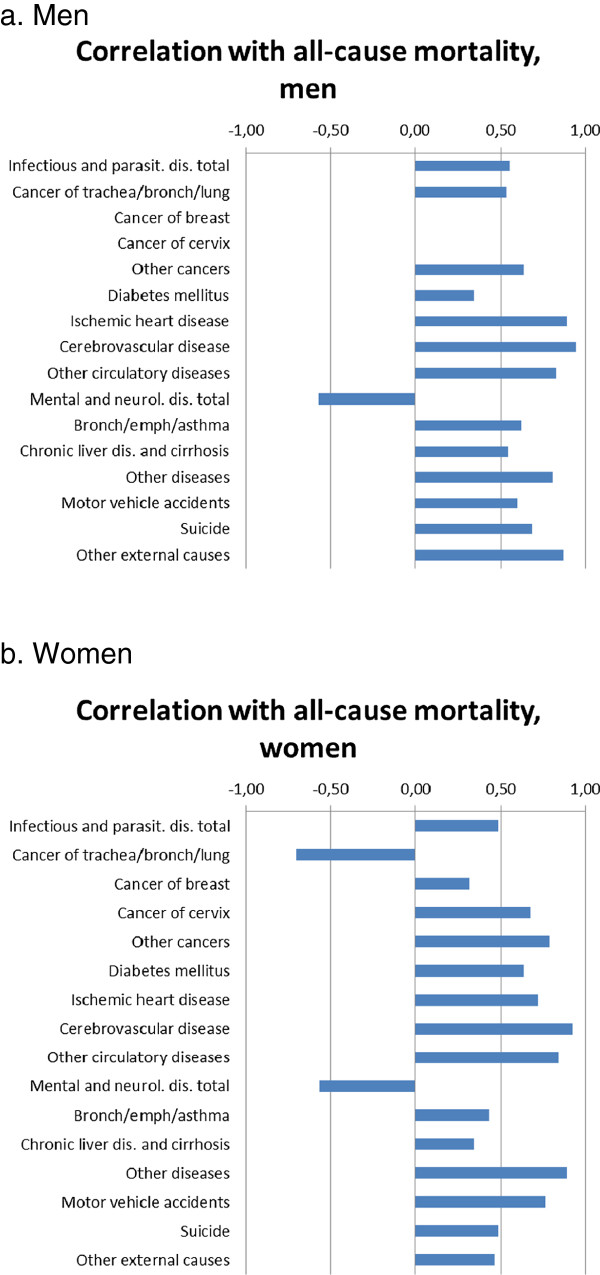
**Correlation between trends in all-cause mortality and trends in cause-specific mortality, European countries, 1970-2009. a**. Men. **b**. Women. Notes: We regressed age-adjusted cause-specific mortality rates and all-cause mortality rates, using ordinary least squares regression, according to a method originally developed by Preston [Preston SH: Mortality patterns in national populations, with special reference to recorded causes of death*.* New York: Academic Press; 1976.] but modified by us by including country dummies to allow for between-country differences in levels of mortality. The graphs present partial correlation coefficients between cause-specific mortality rates on all-cause mortality rates, controlling for country. Full regression results are given in web Additional file 1: Table S1.

It is indeed true that a slight shift in cause-of-death patterns has been occurring in many European countries. Mental and neurological diseases are among the very small number of causes of death for which trends in mortality moved in a different direction as compared to all-cause mortality. While the correlation between trends in all-cause mortality and cause-specific mortality is usually positive, it is negative for mental and neurological diseases (Figure 1; see Additional file 1 for background material to these analyses).

**Figure 2:**
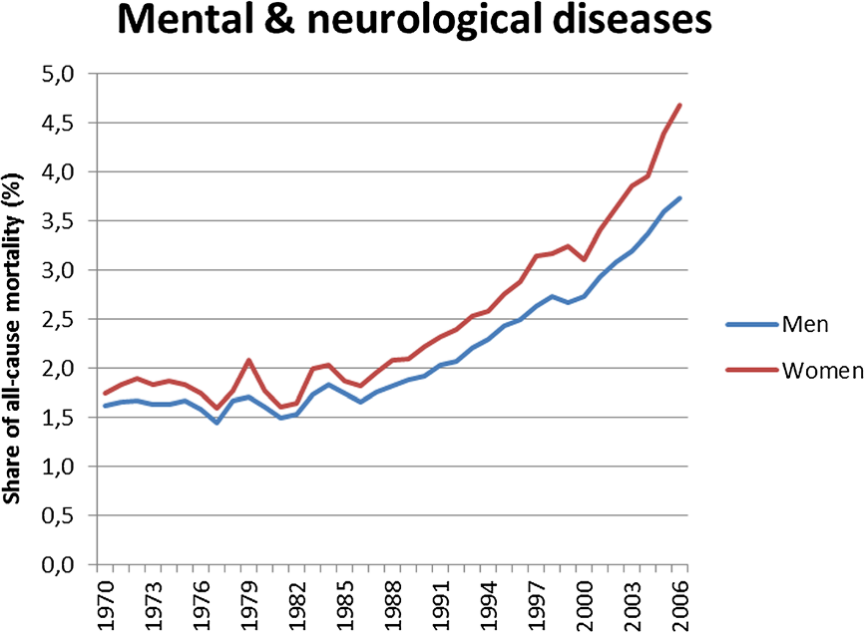
**The rising share of mental and neurological diseases in all-cause mortality, European countries with available data, 1970–2006.** Notes: Average of shares in each European country as calculated on the basis of age-standardized mortality rates. Irregularities around 1979 may be related to the introduction of ICD-9, and irregularities in the early 1980s may be related to the fact that countries in the Soviet Union submitted data for some years, and not for others.

As a result, the average proportion of all-cause mortality that is due to mental and neurological diseases has gone up sharply since the early 1970s (Figure 2). This should not be exaggerated, as the average share of these conditions in all-cause mortality is still below 5%, but it already exceeds 10% among women in several countries. Nevertheless, dementias have recently been reported to be among the top-10 (men) or even top-5 (women) of most frequent causes of death in several high-income countries in Europe and elsewhere [6, 7].

It is unclear what the explanation of these trends is. Previous studies have found that mortality from some mental and neurological diseases has risen, while mortality from other causes in this group has remained stable or has even declined. Over the past decades, many high-income countries have witnessed a rise of age-adjusted mortality from dementia [8]–[11] and motor neuron diseases [12]–[14], but reports on trends in mortality from psychoactive substance use [15]–[18], Parkinson’s disease [14, 19, 20], multiple sclerosis [14, 21, 22] and epilepsy [14, 23, 24] have been less consistent. Mortality from infectious diseases of the central nervous system, such as meningitis, has declined [10].

Even when the direction of the trends is clear, their interpretation is often uncertain, because they are not only determined by trends in incidence and/or survival of these conditions, but also by changes in recognition, diagnosis, certification and coding of causes of death [9, 11, 14, 25, 26]. Over time, the International Classification of Diseases (ICD) has undergone major changes in the chapters of “Mental and behavioural disorders” and “Diseases of the nervous system”. In addition, changes in coding rules have occurred while a single ICD-revision was in use [9, 11, 25, 26].

Nevertheless, it is possible that some of these changes are real, and that some of these conditions actually are more important as causes of death than they were several decades ago. Potential explanations for a real rise of dementia, for example, both as a prevalent condition in the population and as a cause of death, include better survival of people at risk of developing dementia (e.g. better survival of people with risk factors for vascular dementia due to more effective prevention and treatment of ischemic heart disease [27]). Explanatory studies are, however, rare.

We have therefore conducted an in-depth study of trends in mortality from specific mental and neurological conditions in Europe over the past 30 years. The main study questions were:

- Which specific conditions account for the rise of mortality from mental and neurological diseases?
- What were the main variations in these trends between European countries, and what clues do these variations provide for the explanation of the rise of mortality from these conditions?

## Methods

### Data

Mortality data by year (1979–2009), country (n = 41), sex, age (in 5-year age-groups, with 85+ as the highest age-group) and cause of death were extracted from the World Health Organization (WHO) Mortality Data Base (updated November 2012). This dataset contains data on underlying causes of death as officially registered by member states, and as reported to WHO. We restricted the analysis to the post-1979 period because this limited the number of different editions of the International Classification of Diseases (ICD) in use. Due to delays in processing cause-of-death data, the most recent data available for most countries relate to 2009. ICD-codes for the causes of death analysed in this paper are given in web Additional file 2: Table S2, which also includes notes on issues related to cause-of-death classification in this paper.

In addition to the broad chapters of “Mental and behavioural disorders” and “Diseases of the nervous system” we were able to look at seven specific (groups of) causes of death: “dementias” (mainly vascular and unspecified dementias), “mental disorders due to psychoactive substance use” (mainly alcoholic psychosis and alcohol and drug dependence), “meningitis” (some forms of bacterial meningitis not elsewhere classified, i.e. excluding meningococcal meningitis), “Parkinson’s disease”, “Alzheimer’s disease c.a.” (mainly Alzheimer’s disease, but including some other degenerative conditions as well), “multiple sclerosis” and “epilepsy”.

### Analysis

Mortality data were standardized for age (in five-year age-groups until 85+) using the direct method and the European standard population. In a first set of analyses covering all specific conditions we studied all-age mortality, but in subsequent more detailed analyses for dementia we limited the analysis to those aged 65 and over (which also practically eliminates any potential bias due to the inclusion of other hereditary and degenerative diseases of the nervous system).

Only some countries report mortality above the age of 85 with a further distinction by age, and because of the steep gradient of some of these conditions (e.g. dementia) against age we made some additional calculations based on a finer distinction (85–90, 90–94, 95+) for those countries for which this was possible.

We also explored some hypotheses relating to factors that might influence mortality from mental and neurological conditions. We conducted a regression analysis with a forward selection procedure in which we related mortality from mental and neurological conditions to four variables, each representing a different class of background factors:

1. Self-expression values, as an indicator of cultural conditions. We wondered whether health care professionals in populations that adhere more closely to values of self-expression and quality of life instead of mere survival, might be more willing to recognize mental and neurological conditions as a possible cause of death, and/or to refrain from life-saving treatments in dementia patients with low quality of life. People in more advanced industrialized countries have been shown to shift their priorities from basic economic and physical security towards subjective well-being, self-expression and quality of life [28], and these self-expression vs. survival values have been shown to be important determinants of health, health behaviour and health policy in Europe [29, 30]. Data on self-expression vs. survival values (countries’ mean standardized score as measured between 2000 and 2006) were taken from the World Values Survey [28].
2. Average income, as an indicator of economic conditions. Research into the explanation of between-country differences in health indicators often finds that aggregate health outcomes are closely correlated with national income, typically measured by Gross Domestic Product (GDP) [31]. Recent studies have shown that, after a period with diminished influence, average income has again become an important determinant of national mortality levels in Europe [32, 33]. We wondered whether richer countries would, on average, have higher rates of mortality from mental and neurological conditions, for example because they are more advanced in their trajectories of epidemiological transition [34, 35]. Data on national income (in 2008, as measured in 1990 I$1000s) per head of population were extracted from a harmonized dataset compiled by Maddison (http://www.ggdc.net/MADDISON/oriindex.htm) [36].
3. Health care expenditure, as an indicator of health care utilization. Due to its increasing effectiveness in preventing and treating potentially fatal diseases, health care has become a major determinant of mortality in high income countries [37, 38]. We wondered whether countries with a larger health care sector would, on average, have higher mortality from mental and neurological conditions because they have eliminated a larger proportion of potentially amenable mortality [4, 5]. We extracted data on health care expenditure in 2008, as a percentage of Gross Domestic Product, from the World Health Organization Health for All Database (http://data.euro.who.int/hfadb/).
4. Life expectancy at birth. Mortality from many mental and neurological conditions occurs predominantly among elderly people, i.e. among individuals who have escaped death from conditions that kill at younger ages but who because of remaining risk factors or high levels of frailty may be at increased risk of dying from conditions of old age. We therefore wondered whether countries with higher life expectancy at birth would, on average, have higher mortality from mental and neurological conditions [4, 5]. Data on life expectancy at birth in 2008, by gender, were extracted from the Human Lifetable Database (http://www.lifetable.de), supplemented by the World Health Organization Health for All Database (http://data.euro.who.int/hfadb/).

## Results

Table 1 presents some descriptive results for trends in mortality from mental and neurological conditions in Europe. Descriptive results for levels of mortality from these conditions can be found in web Additional file 3: Table S3. For both men and women, the median values (Q2) for changes in mortality from all mental and neurological conditions are positive in all three sub-periods (1981–1991, 1991–2000, 2000–2009) distinguished in this table, implying increases in mortality for a large majority of countries. Variation in mortality change for all mental and neurological conditions between countries is, however, substantial, as indicated by the interquartile range. The values for the first quartile (Q1) are often negative, implying declining mortality in at least a quarter of all countries for which data were available. The values for the third quartile (Q3) are much more strongly positive than the median values, implying very rapid rises in mortality in another group of countries.

**Table 1:** **Change in age-standardized mortality rates for mental and neurological disorders in Europe, 1981 – 2009: median and first and third quartile values, by sex**

		Men				Women			
		1981-91	1991-00	2000-09	1991-2009	1981-91	1991-00	2000-09	1991-2009
**All mental and neurological**	#	18	26	32	26	17	25	32	26
	Q1	-0.69	-0.60	-1.21	-1.39	-0.42	-0.18	0.67	0.92
	Median (Q2)	5.28	2.86	2.61	2.78	1.74	2.48	3.47	7.61
	Q3	9.75	9.20	6.71	8.44	7.69	8.66	7.37	13.15
***Mental and behavioural disorders***	#	27	33	34	33	25	33	34	33
	Q1	-1.64	-1.70	-2.71	-1.69	25	33	34	33
	Median (Q2)	0.92	0.85	-1.11	0.93	0.37	0.40	0.08	0.86
	Q3	3.93	4.00	1.52	5.00	1.31	3.01	1.65	5.79
Dementias	#	17	23	23	20	17	23	26	23
	Q1	0.05	-0.29	-0.54	-0.06	-0.70	-0.25	-0.67	-0.17
	Median (Q2)	0.43	0.26	0.01	2.60	0.69	0.53	0.19	2.40
	Q3	2.36	2.84	2.85	7.10	3.29	3.16	3.26	7.36
Psychoactive substance use	#	17	24	30	24	17	22	25	21
	Q1	-1.16	-1.39	-3.07	-3.67	0.11	-0.46	-0.54	-1.36
	Median (Q2)	0.74	-0.22	-0.89	-1.10	0.54	-0.14	-0.09	-0.38
	Q3	2.87	0.97	-0.01	0.13	0.70	0.20	0.30	0.08
***Diseases of the nervous system***	#	17	24	31	25	17	24	31	25
	Q1	-0.78	0.30	0.69	1.84	-0.48	0.10	0.30	1.41
	Median (Q2)	1.58	2.14	3.08	3.67	0.60	1.89	3.31	3.38
	Q3	3.37	3.28	4.61	7.50	3.02	3.63	4.86	6.38
Meningitis	#	27	32	33	33	27	32	30	30
	Q1	-0.55	-0.28	-0.49	-0.70	-0.50	-0.21	-0.17	-0.33
	Median (Q2)	-0.32	-0.05	-0.28	-0.39	-0.32	-0.11	-0.11	-0.22
	Q3	0.04	0.13	-0.10	-0.21	-0.11	0.06	-0.02	-0.11
Parkinson’s disease	#	17	23	29	25	17	24	30	25
	Median (Q2)	0.41	0.36	0.59	1.23	0.18	0.03	0.24	0.14
	Q3	1.69	0.99	1.82	1.56	1.32	0.50	0.81	0.63
Alzheimer’s c.a	#	17	24	31	25	17	24	31	25
	Q1	0.74	-0.16	-0.36	0.39	0.85	0.19	0.00	1.01
	Median (Q2)	1.74	1.21	1.38	1.75	1.56	1.46	1.01	1.87
	Q3	3.45	1.94	2.55	3.46	3.54	2.39	2.68	4.70
Multiple sclerosis	#	27	30	28	28	27	32	30	29
	Q1	-0.20	-0.18	-0.28	-0.39	-0.30	-0.37	-0.14	-0.33
	Median (Q2)	-0.05	0.03	-0.06	-0.12	-0.02	-0.10	-0.01	0.06
Epilepsy	#	27	32	30	29	27	30	29	29
	Q1	-0.38	-0.61	-0.12	-0.44	-0.41	-0.43	-0.13	-0.38
	Median (Q2)	0.06	-0.12	0.17	-0.07	-0.13	-0.06	0.12	-0.06
	Q3	0.31	0.27	0.32	0.22	0.18	0.14	0.24	0.15

Note: Q1, Q2 and Q3 represent the first quartile, second quartile (or median) and third quartile of the age-standardized mortality rates of all European countries with available data at that point in time. # denotes number of countries in the analysis.

**Figure 3:**
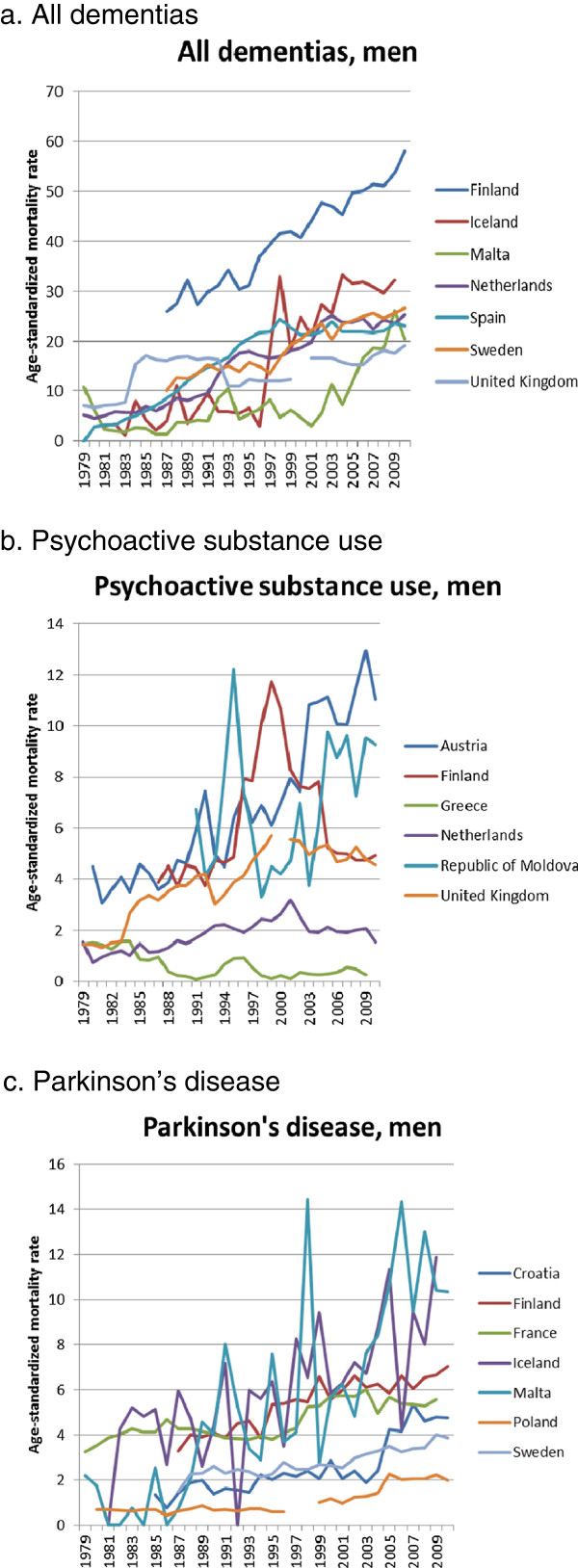
**The rise of age-standardized mortality from (a) all dementias, (b) psychoactive substance use, and (c) Parkinson’s disease, countries in upper quartile of mortality change, men.** Note: Countries in upper quartile of change in age-standardized mortality between 1991 and 2009. Age-standardized mortality rates all ages (in deaths per 100,000 person-years).

**Figure 4:**
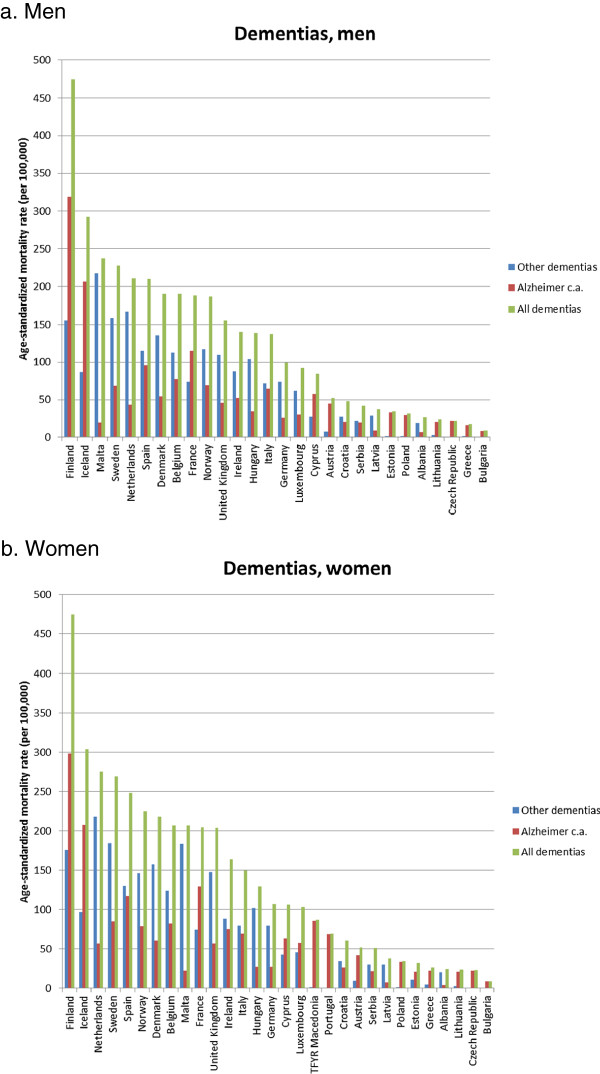
**Age-standardized rates of mortality from all dementias in Europe, 2009, persons aged 65 and over.** **a**. Men. **b**. Women. Note: No deaths from dementia among men reported from Portugal and TFYR Macedonia.

For specific causes of death we also see large variations between countries in mortality trends. For many causes of death increases in some countries go together with declines in others. Median values are consistently positive for dementias, Parkinson’s disease, and Alzheimer’s c.a., implying rising mortality in a majority of countries. Meningitis is the only condition for which median values are consistently negative. For the remaining conditions, median values are sometimes positive, sometimes negative, depending on the time-period. When we focus on the third quartile values, to identify the causes of rapidly rising mortality from all mental and neurological diseases, we find that this must primarily be due to rising mortality from dementias and Alzheimer’s c.a., with some additional but much smaller contribution from Parkinson’s disease (Table 1).Figure 3 illustrates some of the rising mortality trends. In order to allow for possible transfers between causes of death, we combined dementias and Alzheimer’s c.a. in a new group of “all dementias” (Figure 4a). Mortality from this group has increased strongly in some Northern and Western European countries, but also in Malta and Spain. In some countries (e.g. Netherlands, Spain) the trend of mortality from all dementias has recently levelled off, while in others (e.g. Finland, Malta) it continues to rise. Mortality due to psychoactive substance use shows a more erratic pattern, with very strong increases in Austria, and a strong increase followed by a steep decline in Finland (Figure 4b). Mortality from Parkinson’s disease has risen strongly in Iceland and Finland again, but also in Malta and Croatia (Figure 4c).Because of the overwhelming importance of dementias for the rise of mental and neurological diseases the remainder of our analysis focuses on this group of conditions. Figure 4 shows the immense variation in mortality from all dementias between European countries in 2009. The variation is more than 50-fold between the country with the highest mortality rate, Finland, and that with the lowest, Bulgaria. There appears to be a North-west versus South-east gradient, with higher mortality rates in, e.g., most of the Nordic countries and the Netherlands, and lower mortality rates in the Baltic countries and in the Balkans.

**Figure 5:**
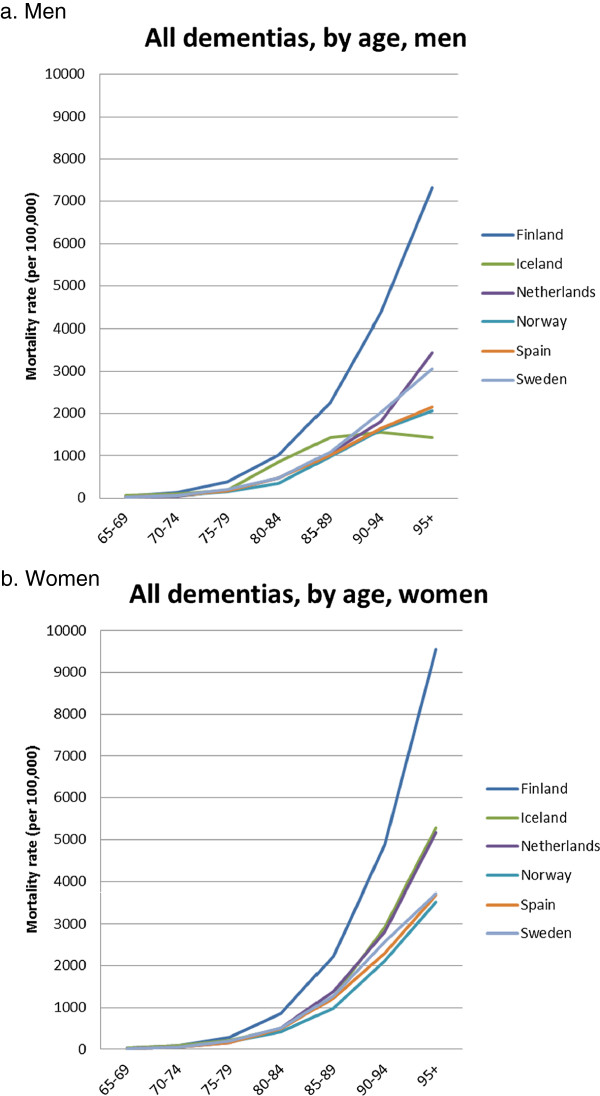
**Age-specific mortality rates from all dementias, countries in upper quartile of mortality, 2009.** **a**. Men. **b**. Women.

For many countries in the upper quarter of the European distribution mortality data are available in a finer breakdown by age, permitting a view on age-specific mortality rates above the age of 85 (Figure 5). In these countries, mortality from all dementias for those above the age of 95 is between 1 and 7% per year among men, and between 3 and 10% per year among women. In these countries, mortality from dementias now accounts for between 5% and 11% of all deaths, and between 9% and 22% of all deaths above the age of 85 (results not shown), and it generally ranks in the top-6 (men) and top-3 (women) of causes of death.

Table 2 shows the results of an exploratory regression analysis. Mortality from all dementias is strongly associated with each of the four variables included in the analysis: self-expression values, average income, health care expenditure and life expectancy at birth, both among men and women. The strongest association is found for self-expression values: the more a national population is oriented towards modern self-expression values, the higher is mortality from all dementias. After controlling for these values, none of the other determinants is statistically significantly associated with mortality from all dementias, while the association with self-expression values mostly remains statistically significant. A backward selection procedure, in which variables are removed one-by-one from a full model (results not shown), leads to the same conclusion: self-expression values are the variable with the strongest association with dementia mortality.

**Table 2:** **Association between mortality from all dementias and potential determinants: results of regression analysis**

		Univariate				Bivariate		
		Beta	Beta	Beta	Beta	Beta	Beta	Beta
		S.E.	S.E.	S.E.	S.E.	S.E.	S.E.	S.E.
**Men**	Self-expression values	54.77				50.34	48.73	54.57
		14.59****				18.53**	28.72	20.15**
	Average income		4.35			0.04		
			1.95**			2.36		
	Health care expenditure			27.55			5.43	
				12.20**			13.60	
	Life expectancy, men				13.84			2.01
					4.38***			8.15
**Women**	Self-expression values	65.19				64.31	52.15	65.16
		14.54****				18.24***	23.89**	19.97***
	Average income		5.10			0.00		
			2.03**			2.33		
	Health care expenditure			27.74			1.07	
				12.66***			12.98	
	Life expectancy, women				27.34			7.84
					7.45***			11.33

Notes: The table shows the regression coefficient (Beta) as well as its standard error (S.E.). In a forward selection procedure, age-standardized mortality from All dementias (aged 65 and over) was regressed on four possible determinants separately (“univariate”), and on self-expression vs. survival values and each of the other possible determinants separately (“bivariate”). Average income: Beta multiplied by 1000 (thus indicating effect of GDP, in 1000I$ per capita, on mortality). *p < .10 **p < .05 ***p < .01 ****p < .01.

## Discussion

### Summary of findings

Rising mortality from mental and neurological conditions is seen in most European countries, and is mainly due to rising mortality from dementias. Mortality from psychoactive substance use and Parkinson’s disease has also risen in several countries. Mortality from dementias has risen particularly strongly in Finland, Iceland, Malta, Netherlands, Spain, Sweden and the United Kingdom, and is positively associated with self-expression values, average income, health care expenditure and life expectancy, but only the first has an independent effect.

### Limitations

This is the most comprehensive study ever of trends in mortality from mental and neurological diseases in Europe, but such wide coverage comes at the expense of relying on official mortality statistics by cause of death. There is no doubt that the conditions studied in this paper increase the risks of dying. For example, the relative risk of dying among dementia patients, as compared to people without dementia of the same age, is between 2 and 3 [39], and that of patients with Parkinson’s disease is between 1.5 and 2.5 [40]. However, their exact role in causing death in individual patients is often less clear, and leaves room for considerable differences in cause-of-death classification.

This has most extensively been studied for dementias. The immediate cause of death of patients with dementia is often pneumonia or cardiovascular disease, and dementia is not always mentioned on death certificates, particularly if death occurs in general hospitals instead of in nursing homes or psychiatric hospitals [41]. Even if dementia is mentioned on the death certificate, it is not always selected as the underlying cause of death, although this has improved with the separate listing of various forms of dementia in ICD-9 and ICD-10 [9] and with changes in coding rules which have more explicitly recognized the possible role of dementia as an underlying cause of death [8, 26, 42]. Underrecording may also apply to Parkinson’s disease [20, 43] or to psychoactive substance use [17]. This has led some researchers to combine dementia coded as an underlying cause with dementia coded as a contributory cause [8, 44]–[46]. This approach certainly removes some of the underrecording of dementia and has been shown to correctly identify a high proportion of dementia cases [47, 48]. It would therefore be interesting to compare our results with those from an analysis based on all mentions of dementia on death certificates, but this was not feasible in the study reported in this paper as the WHO Mortality Data Base only contains information on underlying causes of death. The implication is that our study is likely to underestimate the role of dementia as a cause of death, and because this underestimation is likely to also differ between countries, we must refrain from substantive interpretations of the between-country variations seen in our study.

Studies of time-trends in the incidence of dementia and other mental and neurological conditions are scarce. The limited evidence-base, however, does not suggest a rise in the incidence in these conditions. Community studies in the United States have not detected clear time trends in the incidence of dementia [27, 49], while a study from the same country based on medical records reported an increase [50]. Community studies from the Netherlands and England even found declines in the incidence or prevalence of dementia [51, 52]. Incidence or prevalence of Parkinson’s disease have probably not been rising either [53]. With regard to psychoactive substance use, the available data show great between-country variability in trends in alcohol consumption, with rising trends in, e.g., Finland and the United Kingdom (cf. Figure 3b), and a lack of comparative data on trends in other health outcomes than mortality [54].

It is unlikely, therefore, that the increase in mortality from dementia as found in many European countries represents an increase in incidence of the disease. As it is also unlikely that survival of patients with dementia has declined substantially, this leaves improved recognition, diagnosis, certification and/or coding as the most likely explanation for the increase in mortality from dementia. The same may apply to Parkinson’s disease.

An exploration of the association between mortality from dementias and from “senility”, a now obsolete category in the International Classification of Diseases that may have contained many cases of unrecognized dementia [55], shows that national levels of mortality from both conditions were indeed negatively correlated throughout the study period, but not very strongly so (web Additional file 4: Table S4). In some countries, such as Spain and Estonia, declining mortality from senility coincided with increasing mortality from dementias, but in others, such as Finland and Bulgaria, the trends do not suggest a transfer of deaths from one to the other category (web Additional file 5: Figure S1).

### Interpretation

Changes in recognition, diagnosis, certification and/or coding of dementia also reflect changes in physicians’ and statisticians’ attitudes, in this case towards the possible role of dementia as a cause of death. Our findings on the association between cultural values and mortality rates from dementia should probably be interpreted in this sense.

We hypothesized that health care professionals in populations that adhere more closely to values of self-expression and quality of life instead of mere survival, are more willing to recognize mental and neurological conditions as a possible cause of death, and/or to refrain from life-saving treatments in dementia patients with low quality of life. Our results can be seen to support this hypothesis: mortality from dementias is indeed higher in countries scoring higher on the self-expression versus survival values scale. This can probably be explained from the fact that a greater emphasis on quality of life as the main purpose of health care interventions may first lead to a greater awareness of dementia and other non-directly fatal diseases for elderly patients’ well-being, and then to a greater recognition of these conditions as the ultimate causes of their patients’ deaths. We have recently demonstrated that self-expression values are strong predictors of between-country variations in a wide range of health-related behaviours [30].

One could perhaps even raise the issue of whether rapidly rising rates of mortality from dementia indicate a greater willingness of health care professionals to refrain from life-saving treatment in the face of a debilitating illness. As long as its complications are adequately treated, dementia will in itself not lead to death, so when a death is certified as caused by dementia, the physician has probably decided that further treatment was useless [56]. Some of the countries with the highest mortality rates from dementia (e.g., Sweden, Netherlands, Denmark, Belgium) are among the countries with the highest public acceptance rates of euthanasia, and acceptance rates of euthanasia are also closely associated with “modern” value orientations [57]. On the other hand, the correlation between the two phenomena is far from perfect, as illustrated by Malta which combines very high mortality from dementia with very low public acceptance of euthanasia [57]. Euthanasia practice is also not necessarily more liberal in the North of Europe than in other parts of the subcontinent [58]. It is therefore more likely that rising rates of dementia more simply reflect the greater recognition of the debilitating nature of this disease that comes with modern shifts in value orientations.

## Conclusions

Although trends in mortality from dementias have probably been affected by changes in cause-of-death classification, the high level of mortality from these conditions in a number of vanguard countries suggests that it is now among the most frequent causes of death in high-income countries. Recognition of dementias as a cause of death, and/or refraining from life-saving treatment for patients with dementia, appear to be strongly dependent on cultural values.

## Electronic supplementary material

Additional file 1: Table S1: Regression of cause-specific mortality on all-cause mortality, Europe, 1970-2009. Notes: Age-adjusted mortality rates were extracted from the World Health Organization Health for All Database Data (http://data.euro.who.int/hfadb/). We imputed some missing data using information from adjacent years and/or adjacent countries, and then redistributed “signs, symptoms and ill-defined conditions” proportionally over all specific causes of death (excluding injuries). In order to assess whether mortality trends for specific causes paralleled all-cause mortality trends we regressed cause-specific mortality rates on all-cause mortality rates, using ordinary least squares regression. This method was originally developed by Preston [Preston SH: Mortality patterns in national populations, with special reference to recorded causes of death. New York: Academic Press; 1976.] but modified by us by including country dummies to allow for between-country differences in levels of mortality. Partial correlation coefficients calculated according to A. Gelmann & J. Hill 2007: Data Analysis Using Regression and Multilevel/Hierarchical Models, p.474. (DOCX 29 KB)

Additional file 2: Table S2: ICD-codes and notes related to cause-of-death classification. (DOCX 29 KB)

Additional file 3: Table S3: Age-standardized mortality rates for mental and neurological disorders in Europe, 1981 – 2009: median and first and third quartile values, by sex. (DOCX 33 KB)

Additional file 4: Table S4: Correlation between age-standardized mortality rate for All dementias and for senility, 1980, 1990, 2000 and 2009, by sex. (DOCX 25 KB)

Additional file 5: Figure S1: Trends in mortality from All dementias and from senility, selected countries, by sex. (DOCX 101 KB)
