# Supplementary material for: The rise of mortality from mental and neurological diseases in Europe, 1979–2009: observational study
Source: BMC Public Health. 2014 Aug 13;14:840. doi: 10.1186/1471-2458-14-840 (PMC4139616; doi:10.1186/1471-2458-14-840)
Supplement: Supplementary file 1 — Additional file 1: Table S1: Regression of cause-specific mortality on all-cause mortality, Europe, 1970-2009. Notes: Age-adjusted mortality rates were extracted from the World Health Organization Health for All Database Data (http://data.euro.who.int/hfadb/). We imputed some missing data using information from adjacent years and/or adjacent countries, and then redistributed “signs, symptoms and ill-defined conditions” proportionally over all specific causes of death (excluding injuries). In order to assess whether mortality trends for specific causes paralleled all-cause mortality trends we regressed cause-specific mortality rates on all-cause mortality rates, using ordinary least squares regression. This method was originally developed by Preston [Preston SH: Mortality patterns in national populations, with special reference to recorded causes of death. New York: Academic Press; 1976.] but modified by us by including country dummies to allow for between-country differences in levels of mortality. Partial correlation coefficients calculated according to A. Gelmann & J. Hill 2007: Data Analysis Using Regression and Multilevel/Hierarchical Models, p.474. (DOCX 29 KB) [file 12889_2013_6960_MOESM1_ESM.docx]

**Table A1. Regression of cause-specific mortality on all-cause mortality, Europe, 1970-2009.**

Notes: Age-adjusted mortality rates were extracted from the World Health Organization Health for All Database Data (<http://data.euro.who.int/hfadb/>). We imputed some missing data using information from adjacent years and/or adjacent countries, and then redistributed “signs, symptoms and ill-defined conditions” proportionally over all specific causes of death (excluding injuries). In order to assess whether mortality trends for specific causes paralleled all-cause mortality trends we regressed cause-specific mortality rates on all-cause mortality rates, using ordinary least squares regression. This method was originally developed by Preston ([59](#_ENREF_59)) but modified by us by including country dummies to allow for between-country differences in levels of mortality. Partial correlation coefficients calculated according to A. Gelmann & J. Hill 2007: Data Analysis Using Regression and Multilevel/Hierarchical Models, p.474.
