# Supplementary material for: The rise of mortality from mental and neurological diseases in Europe, 1979–2009: observational study
Source: BMC Public Health. 2014 Aug 13;14:840. doi: 10.1186/1471-2458-14-840 (PMC4139616; doi:10.1186/1471-2458-14-840)
Supplement: Supplementary file 2 — Additional file 2: Table S2: ICD-codes and notes related to cause-of-death classification. (DOCX 29 KB) [file 12889_2013_6960_MOESM2_ESM.docx]

**Table A2. ICD-codes and notes related to cause-of-death classification**

ICD-9 generally came in use around the year 1979, and ICD-10 in the late 1990s, but with considerable variation between countries. For example, Denmark never used ICD-9, and went directly from ICD-8 to ICD-10. During the period in which ICD-9 was in use, countries reported their mortality data to WHO according to a so-called Basic Tabulation List, which has less detail than the original version of ICD-9. The Soviet Union used a modification of ICD-9. Many countries of the former Soviet Union and Switzerland do not use the original version of ICD-10, but use a less detailed version (denoted as ICD-10(1)). ICD-codes for the causes of death analysed in this paper are given in the following table.

In 2009 and for countries with complete data, the two analysed conditions (dementias and mental health disorders due to psychoactive substance use) on average cover 90% (standard deviation (SD) 7%) of all deaths in the chapter “Mental and behavioural disorders” among men, and 87% (SD 17%) of all deaths in this chapter among women. The five analysed conditions in the chapter “Diseases of the nervous system” (meningitis, Parkinson’s disease, Alzheimer’s disease, multiple sclerosis and epilepsy) on average cover 72% (SD 7%) of all deaths in this group among men, and 75% (SD 5%) of all deaths in this group among women.

Both in ICD-9 and ICD-10 cerebrovascular disease was classified in the broad chapter of “Diseases of the circulatory system”, and therefore excluded from all our analyses, but in ICD-10 transient ischemic attacks (G45) were part of the chapter “Diseases of the nervous system” and included in that category in our analyses. The largest classification problem that we were unable to solve is that in the Basic Tabulation List of ICD-9 Alzheimer’s disease was grouped together with some other hereditary and degenerative diseases of the nervous system, including Huntington's disease, spinocerebellar disease and motor neuron disease. These are relatively rare conditions, but this implies that we have overestimated mortality from Alzheimer’s disease during the years in which ICD-9 was in use, and that we have underestimated the rise of Alzheimer’s disease since then.
