# Supplementary material for: The rise of mortality from mental and neurological diseases in Europe, 1979–2009: observational study
Source: BMC Public Health. 2014 Aug 13;14:840. doi: 10.1186/1471-2458-14-840 (PMC4139616; doi:10.1186/1471-2458-14-840)
Supplement: Supplementary file 3 — Additional file 3: Table S3: Age-standardized mortality rates for mental and neurological disorders in Europe, 1981 – 2009: median and first and third quartile values, by sex. (DOCX 33 KB) [file 12889_2013_6960_MOESM3_ESM.docx]

**Table A3. Age-standardized mortality rates for mental and neurological disorders in Europe, 1981 – 2009: median and first and third quartile values, by sex**

Note: Q1, Q2 and Q3 represent the first quartile, second quartile (or median) and third quartile of the age-standardized mortality rates of all European countries with available data at that point in time. # denotes number of countries in the analysis.
