# Supplementary material for: The rise of mortality from mental and neurological diseases in Europe, 1979–2009: observational study
Source: BMC Public Health. 2014 Aug 13;14:840. doi: 10.1186/1471-2458-14-840 (PMC4139616; doi:10.1186/1471-2458-14-840)
Supplement: Supplementary file 4 — Additional file 4: Table S4: Correlation between age-standardized mortality rate for All dementias and for senility, 1980, 1990, 2000 and 2009, by sex. (DOCX 25 KB) [file 12889_2013_6960_MOESM4_ESM.docx]

**Table A4. Correlation between age-standardized mortality rate for All dementias and for senility, 1980, 1990, 2000 and 2009, by sex**

Note: Code for senility is B465 in ICD-9(BTL), and R54 in ICD-10.
