# Supplementary material for: The rise of mortality from mental and neurological diseases in Europe, 1979–2009: observational study
Source: BMC Public Health. 2014 Aug 13;14:840. doi: 10.1186/1471-2458-14-840 (PMC4139616; doi:10.1186/1471-2458-14-840)
Supplement: Supplementary file 5 — Additional file 5: Figure S1: Trends in mortality from All dementias and from senility, selected countries, by sex. (DOCX 101 KB) [file 12889_2013_6960_MOESM5_ESM.docx]

**Figure A1. Trends in mortality from All dementias and from senility, selected countries, by sex**

1. Finland


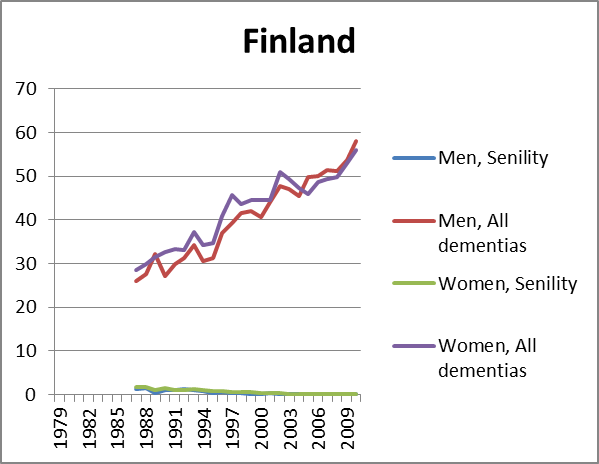


1. Spain


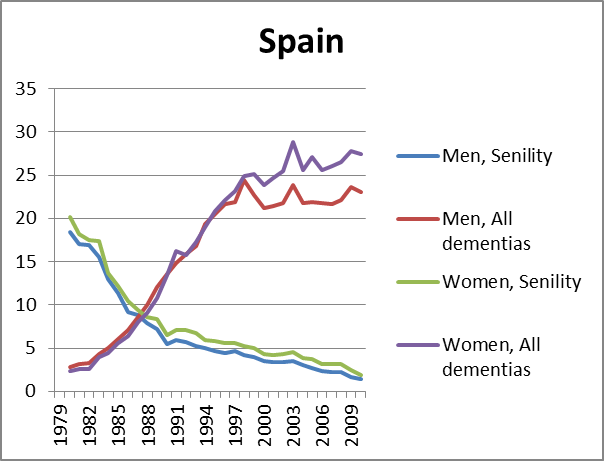


1. Estonia


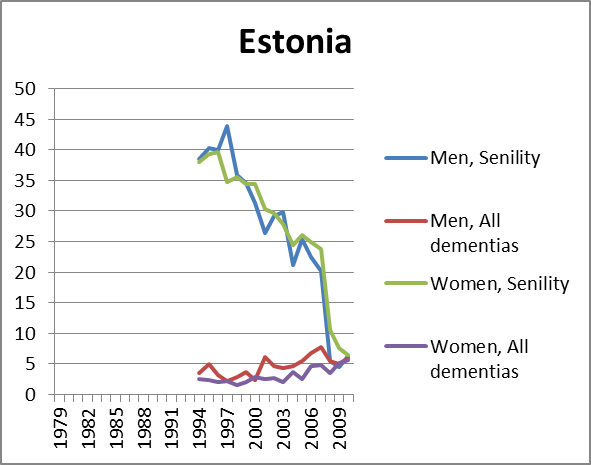


1. Bulgaria


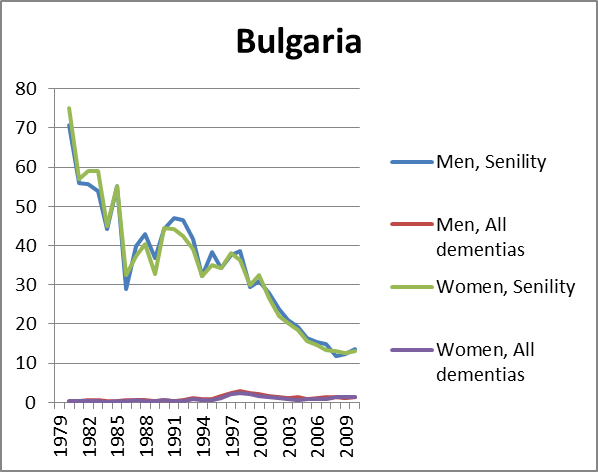


Note: Code for senility is B465 in ICD-9(BTL), and R54 in ICD-10.
